# Patients with Intolerance Reactions to Total Knee Replacement: Combined Assessment of Allergy Diagnostics, Periprosthetic Histology, and Peri-implant Cytokine Expression Pattern

**DOI:** 10.1155/2015/910156

**Published:** 2015-03-19

**Authors:** Peter Thomas, Christine von der Helm, Christoph Schopf, Farhad Mazoochian, Lars Frommelt, Hans Gollwitzer, Josef Schneider, Michael Flaig, Veit Krenn, Benjamin Thomas, Burkhard Summer

**Affiliations:** ^1^Klinik und Poliklinik für Dermatologie und Allergologie der Ludwig-Maximilians-Universität (LMU), Frauenlobstraße 9-11, 80337 München, Germany; ^2^Klinik und Poliklinik für Orthopädie der LMU, Marchioninistraße 15, 81377 München, Germany; ^3^Endoklinik, Abteilung für Mikrobiologie, Holstenstraße 2, 22767 Hamburg, Germany; ^4^Klinik für Orthopädie und Sportorthopädie der Technischen Universität München (TU), Ismaninger Straße 22, 81675 München, Germany; ^5^Institut für Pathologie, Max-Planck-Straße 5, 54296 Trier, Germany

## Abstract

We performed a combined approach to identify suspected allergy to knee arthroplasty (TKR): patch test (PT), lymphocyte transformation test (LTT), histopathology (overall grading; T- and B-lymphocytes, macrophages, and neutrophils), and semiquantitative Real-time-PCR-based periprosthetic inflammatory mediator analysis (IFN*γ*, TNF*α*, IL1-*β*, IL-2, IL-6, IL-8, IL-10, IL17, and TGF*β*). We analyzed 25 TKR patients with yet unexplained complications like pain, effusion, and reduced range of motion. They consisted of 20 patients with proven metal sensitization (11 with PT reactions; 9 with only LTT reactivity). Control specimens were from 5 complicated TKR patients without metal sensitization, 12 OA patients before arthroplasty, and 8 PT patients without arthroplasty. Lymphocytic infiltrates were seen and fibrotic (Type IV membrane) tissue response was most frequent in the metal sensitive patients, for example, in 81% of the PT positive patients. The latter also had marked periprosthetic IFN*γ* expression. 8/9 patients with revision surgery using Ti-coated/oxinium based implants reported symptom relief. Our findings demonstrate that combining allergy diagnostics with histopathology and periprosthetic cytokine assessment could allow us to design better diagnostic strategies.

## 1. Introduction

Hip and knee replacement are very successful surgical procedures to restore quality of life in osteoarthritis patients [1] and correspondingly implantation rates are steadily increasing [2]. However, in a substantial part of such patients implant failure leads to implant revision. A recent review lists a total knee replacement (TKR) revision rate of 9.5% in Germany and of 8.4% in the USA for the year 2011 [2]. Within the spectrum of conditions leading to TKR failure [3] adverse reactions may be found, but the role of allergy is still a controversial issue. Cutaneous metal allergy is frequent in the general population, for example, approximately 13% to nickel (Ni), 2% to cobalt (Co), and 1% to chromium (Cr) [4]. Either wear or corrosion leads to peri-implant and systemic metal (particularly Ni, Cr, or Co) exposure of arthroplasty patients [5], and correspondingly, prevalence of metal sensitivity in patients with failed implant is reported to be increased [6–8]. With regard to total hip replacement, aseptic loosening was mostly attributed to wear associated macrophage dominated foreign body response as underlying mechanism [9, 10]. Over the last decade, attention has turned to the role of hypersensitivity in peri-implant inflammation. In particular in metal-on-metal arthroplasty a subgroup of patients was described showing rather peri-implant lymphocytic inflammation as potential elicitor of implant failure [11–13]. Described histologic changes include diffuse, perivascular or lymph-follicle (lymphoid) like infiltration of lymphocytes and few macrophages, high endothelial venules, and in part tissue necrosis [13–15]. Metal allergy as a contributing factor was suggested by the observed linkage between peri-implant lymphocytic inflammation, patch test reactivity to metals, and enhanced lymphocyte transformation test (LTT) response to metals in a series of such patients [16]. There is however still controversial discussion, as to which extent metal allergy contributes to the “umbrella term” adverse reactions in hip arthroplasty [17, 18]. In fact, metal allergic patients may well tolerate the respective metals containing implant [8, 19]. With regard to TKR there also exist reports on cutaneous metal allergy found in association with complications [20, 21], but it is not yet further investigated whether such metal allergy is responsible for the biological reaction. As we are seeing patients with suspected implant intolerance reactions in a special outpatient ambulatory, we wondered whether in a series of patients with complicated TKR a potential metal sensitivity could be linked to a particular peri-implant histological picture and cytokine expression pattern. To address potential clinical relevance of the findings we further contacted the patients to see if they have had some benefit from revision surgery with particular attention to the potential use of “hypoallergenic” materials upon revision surgery.

Thus, the aim of the investigation was to better prove allergic etiology in complicated TKR by combining different diagnostic steps and by assessing the clinical relevance of the findings in a followup.

## 2. Materials and Methods

### 2.1. Patients and Controls

A total of* 45 patients* took part in the study. The study was approved by the ethics committee. The following patient groups were analyzed.

*25 knee arthroplasty patients* (16 m, 9 f, 37–75 years) with CoCrMo based TKR and complications like loosening, recurrent effusions, and pain were presented by their treating orthopaedic surgeons to our ambulatory for allergy diagnostics, since preceding diagnostics gave no indication of problem elicitors like malpositioning/malalignment, fracture, or infection. According to the allergy diagnostics results, these patients were further assigned to three groups.* Group I*: 11 patients (patch test positive and LTT positive),* Group II*: 9 patients (patch test negative and LTT positive), and Group III: 5 patients (patch test negative and LTT negative).

The study included* 12 patients* (1 m, 52–89 years; “*OA-control group*”) without implant, but degenerative joint disease/osteoarthritis (OA) prior to knee arthroplasty.

The study included* 8 patients* (2 m, 53–75 years; “*PT-control group*”) without implant, but having undergone patch test (PT) for suspected allergic skin diseases. 6/8 had Ni-PT reactivity, 2/8 had no Ni-PT reactivity.

In the 25 TKR patients, potential metal sensitivity was assessed by PT and LTT; furthermore, periprosthetic tissue samples were obtained for histology, molecular analysis, and microbiology. In addition a WOMAC score was obtained at the ambulatory visit to have feedback on the patients' perception of daily life activity and of pain. In the 12 OA patients (“*OA-control group*”), at primary TKR tissue samples were obtained for histology, molecular analysis, and microbiology. In the 8 “PT-*control group*” patients, biopsies were obtained from the 6 Ni-PT reactive and the 2 Ni-non reactive PT areas for histology and molecular analysis. The characteristics of the 45 patients are summarized in Table 1.

### 2.2. Patch Test (PT)

In the 25 TKR patients an European standard series of 30 contact allergens including a Co, a Cr, and a Ni preparation (Hermal, Reinbek, Germany) supplemented by a metal allergen series (Brial Allergen GmbH, Greven, Germany) as well as a bone cement component series in case of cemented arthroplasty was tested on the patients' back. Test preparations were applied in Finn chambers for 2 days and reactions were evaluated on the day of removal and at 3 days after application. In the patients with bone cement series testing, an additional reading was performed after 1 week. Grading of the skin reactions was as recommended by the German Contact Dermatitis Research Group.

### 2.3. Lymphocyte Transformation Test (LTT)

Peripheral blood mononuclear cells (PBMC) were obtained from the heparinized blood of the TKR patients by density centrifugation on Ficoll-Hypaque (Phadia, Freiburg, Germany). Cells (1 × 10^6^/mL) were cultured in RPMI1640 medium (Biochrom, Berlin, Germany) supplemented by autologous serum, glutamine, antibiotic-antimycotic-solution, and nonessential amino acids. All cultures were performed in quadruplicate in 96-well plates (Nunc, Roskilde, Denmark). Stimuli were the pan T-cell mitogen phytohemagglutinin (PHA) 2.4 *μ*g/mL, tetanus toxoid (TT) 5 *μ*g/mL, NiSO_4_, CrCl_3_, and CoCl_2_ (7 concentrations each from 1 × 10^−4^ M to 1 × 10^−6^ M) and culture medium alone as control. After 5 days, cells were pulsed with ^3^H thymidine overnight and proliferation was assessed by incorporated radioactivity. The stimulation index (SI) was calculated by the ratio of mean counts per minute (cpm) of stimulated to unstimulated cultures. SI > 3 was considered as positive.

### 2.4. Analysis of Peri-Implant Tissue

In the 25 TKR patients, tissue specimens were obtained from the newly formed articular capsule at the time of revision. At least 2 probes were sent for microbiological evaluation to the Endoklinik Hamburg. The other two probes were processed for histology and one probe for molecular analysis. In the 12 OA-patients tissue specimens were obtained at the moment of primary arthroplasty implantation for histology and molecular analysis as above. The 8 “*PT-control group*” patients underwent punch biopsy of their Ni-PT areas on the back after test reading on D3. One probe each was obtained for histology and molecular analysis.

### 2.5. Histological Examination

The formalin-fixed tissues were processed and stained with haematoxylin-eosin. Immunohistology was performed with antibodies to T-cell (*α*CD3), B-cell (*α*CD20), macrophage (*α*CD68 resp KP1), and neutrophil (*α*CD15) antigens. The sections were microscopically examined and the proportionate distribution of the tissue components including macrophages, diffuse or perivascular accumulation of T- or B-lymphocytes, and plasma cells as well as the overall reaction pattern of the tissue specimen were semiquantitatively assessed. The grading score was according to Krenn et al. [24] and in the case of the TKR-patient derived samples the consensus classification [24] was used. This consensus classification does subdivide the peri-implant tissue reaction patterns into a particle dominated foreign body like response (Type I), a granulocyte dominated infectious type (Type II), the mixture of Types I and II (combined Type, Type III), and a paucicellular and rather fibrotic reaction (Type IV, indifferent type).

### 2.6. Molecular Analysis

The following probes were obtained in RNA-conserving liquid for subsequent analysis: from each of the 25 TKR patients and 12 OA-control patients peri-implant and subcutaneous tissue (reference) probe; from the patch test control group 6 Ni-PT-positive probes and 2 probes from Ni-non-reactive test site (reference).

Total RNA was isolated from the tissue specimen by phenol/chloroform extraction and reverse transcribed into cDNA by the use of AMV reverse transcriptase. The expression of the following cytokines was analysed by semiquantitative RT-PCR in the LightCycler: IFN*γ*, TNF*α*, IL1-*β*, IL-2, IL-6, IL-8, IL-10, IL17, and TGF*β*. The expression value was determined in comparison to the house-keeping gene EF1-*α* [25] by the ΔΔCt-method by Schmittgen and Livak [22].

### 2.7. Comparison of Pre- and Postoperative WOMAC Score

A modified score system has been used in accordance with the publication of Roos et al. [23]. As from the Groups I and II patients preoperative WOMAC knee score was available, we further contacted patients after revision surgery to also get (at not less than 6 months after surgery) their postoperative WOMAC score information. Particular focus was put on patients with revision by use of “hypoallergenic” material.

## 3. Results and Discussion

Metal implant allergy still remains a diagnosis of exclusion, with a delay in diagnosis due to missing disease specific criteria and need of combining different diagnostic steps. Thus various complication elicitors are questioned first in TKR failure and metal implant allergy is diagnosed by a combination of PT, LTT, and histopathology [26, 27]. The study presented here aims to support improvement of diagnostic tools.

### 3.1. Allergy Diagnostics

Within the 20 patients with metal sensitivity, 11 (Group I) showed PT reactions to metals (in part multiple reactions): 10 to Ni, 6 to Co, 2 to Cr, and one of these 11 patients also showed PT reactions to bone cement components (to gentamicin and benzoyl peroxide). In 6/11 additional LTT data were available and showed 5x Ni reactivity and 1 nonreactive LTT. In the remaining 9 patients (Group II) with negative PT to implant components but positive LTT we found 9x LTT reactivity to Ni, 1x to Co. These data are summarized in Tables 2–4. In several studies increased metal sensitivity has been found in patients with arthroplasty [6, 7]. At a larger scale, when comparing 100 symptom-free to 200 complicated arthroplasty patients, such increased incidence of metal allergy—in particular to Ni—could be reproduced [21]. Most data on in vivo metal release conditions are derived from hip arthroplasty patients. However local Co and Cr release is seen also in TKR and respective systemic exposure of the patients is reported [28]. Furthermore, also substantial Ni release might be observed in CoCrMo-arthroplasty patients [29]. The predominance of Ni allergy in our patient groups might thus not only reflect relative predominance of Ni allergy in the general population. On the other hand, even symptom-free patients with well performing knee arthroplasty may have cutaneous metal allergy to implant alloy metals [8, 19]. Thus, as Granchi et al. stated in 2012 [30] that presence of metal sensitivity may not mean implant failure mechanism at the single patient level.

### 3.2. Histological Examination

**Figure 1:**
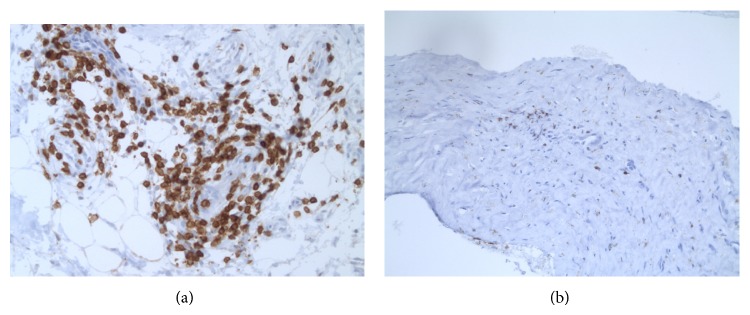
(a) Example of perivascular lymphocytic infiltrate; *α*CD3 stain. (b) Example of scattered periprosthetic lymphocytes; *α*CD3 stain.

**Figure 2:**
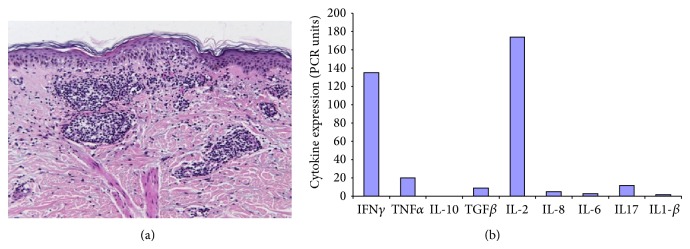
(a) Histology of patch test (PT) reaction to Ni showing perivascular T-lymphocytic infiltrates, scattered eosinophils, and epidermal “spongiotic” changes. (b) Relative inflammatory mediator expression in biopsy of positive PT reaction to Ni. Analysis based on semiquantitative real-time RT-PCR.

We next wondered if the periprosthetic tissue analysis would help to discriminate hyperergic tissue response. For this purpose, four conditions were chosen for comparative analysis of tissue specimen. For example, periprosthetic tissue samples were obtained from (1) the 20 TKR patients with complications and metal sensitivity (Groups I and II); (2) the 5 TKR patients with complications but no metal sensitivity (Group III); (3) 12 patients with degenerative knee joint disease/osteoarthritis (OA-control group) at primary arthroplasty; and (4) the cutaneous biopsies that were performed at PT sites in 8 PT patients (PT-control group) of whom 6 had positive, eczematous PT reaction to Ni and 2 had no PT reaction to Ni. The rating of periprosthetic/(neo) capsule tissue response was done according to the standardized consensus classification initially published by Morawietz et al. in 2006 [31] and revised by Krenn et al. [24]. In addition focus was put on the presence of T-lymphocytes, B-lymphocytes, neutrophils, and macrophages—and furthermore probes of Groups I, II, and III patients were also sent to microbiology evaluation. Several unexpected findings were made: 9/11 patients in Group I and 6/9 patients in Group II had a collagen fibre rich, connective tissue resembling periprosthetic tissue reaction (Type IV/indeterminate type). And only 5 of the 20 metal sensitive patients had the overall picture of the “wear particle induced type” with macrophage dominated response. This is in contrast to the general observation of mostly wear particle/foreign body response like tissue pattern in failed arthroplasty and to the only 15% Type IV (fibrotic) response reactivity in the 370 samples analysed by Morawietz et al. [31]. There were no signs of infections in these 20 samples of our Groups I and II patients. Despite being a predominant “arthrofibrosis”-like, paucicellular reactivity, presence of lymphocytes was noted in perivascular or scattered distribution (Figures 1(a) and 1(b)). In contrast, out of the 5 patients without metal sensitivity two showed infection and lymphocytic inflammation was only seen in one of these patients. In OA-patients, again, lymphohistiocytic infiltrates were noted together with absence of neutrophils. These findings are summarized in Tables 2, 3, and 4. Figures 1(a) and 1(b) are representative histology findings of patients in Groups I and II. Biopsies from Ni-induced allergic patch test reactions were characterized by perivascular and sometimes diffuse lymphohistiocytic infiltrates together with contact allergy-typical epidermal changes as shown in a representative sample (Figure 2(a)). Witzleb et al. speculated that perivascular or diffuse presence of (T-)lymphocytes in periprosthetic tissue could be interpreted as hyperergic response [15]. However, von Domarus and coworkers [32] described T lymphocyte infiltration as a common finding in tissue samples of retrieved aseptically loosened metal-on-polyethylene arthroplasties. Thus, they conclude that neither necrobiosis nor infiltration of T-lymphocytes should be considered to be specific for metal hypersensitivity reaction.

### 3.3. Cytokine Expression Profile

**Figure 3:**
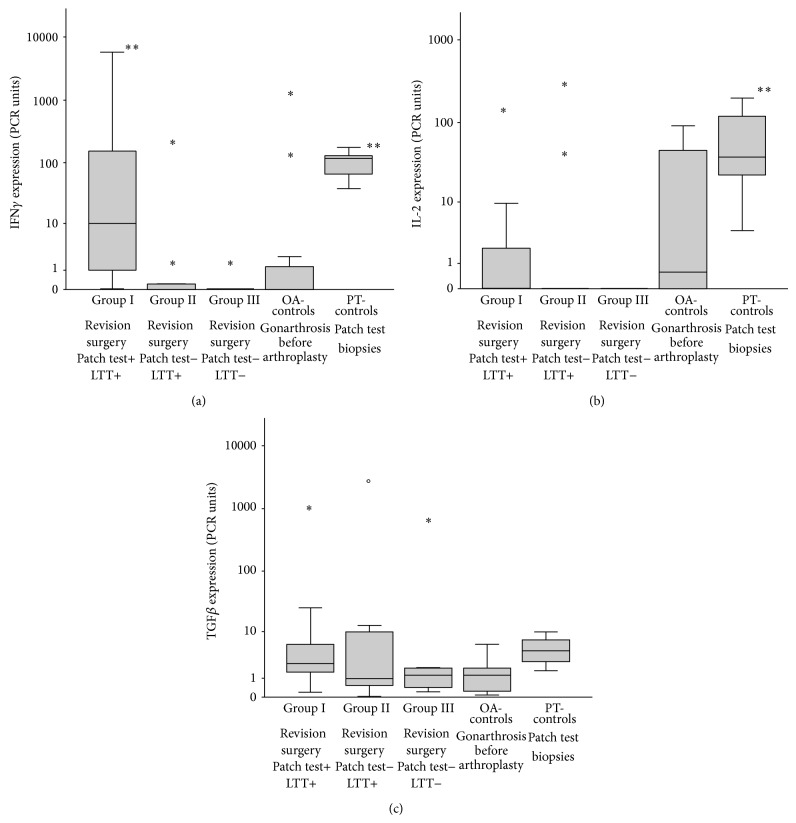
(a) Cytokine expression of IFN*γ* in the tissues of the 5 different patient groups; cytokine expression was analysed in comparison to the house-keeping gene EF-1*α* and to the patients control tissue by the ΔΔCt-method [22];  ^**^ = *P* < 0.005 (Wilcoxon-Mann-Whitney-test done by SPSS statistical software). (b) Cytokine expression of IL-2 in the tissues of the 5 different patient groups; cytokine expression was analysed in comparison to the house-keeping gene EF-1*α* and to the patients control tissue by the ΔΔCt-method [22];  ^**^ = *P* < 0.005 (Wilcoxon-Mann-Whitney-test done by SPSS statistical software). (c) Cytokine expression of TGF*β* in the tissues of the 5 different patient groups; cytokine expression was analysed in comparison to the house-keeping gene EF-1*α* and to the patients control tissue by the ΔΔCt-method [22] (Wilcoxon-Mann-Whitney-test done by SPSS statistical software).

In view of the partly inconclusive publications [27, 32–34] we next wondered whether assessment of inflammatory mediator expression could improve characterization of the tissue response pattern. In Figure 2(b) the cytokine RNA expression pattern of an acute ongoing specific cutaneous delayed type hypersensitivity reaction to Ni is shown. Major findings are the marked upregulation of IFN*γ* as typical marker of the Th1-response stimulating the cellular immune response and of IL-2 indicating T-cell activation and proliferation [35]. When assessing the groups of metal TKR patients with/without metal sensitivity and OA-control group, such upregulation of IFN*γ* was also particularly visible in Group I patients, for example, TKR with complications and patch test reactivity to metals. Out of the other mediators assessed, in the TKR patients IL-2 expression was more prominent in Group I and in OA-patient Group - and TGF-ß expression slightly more in Groups I and II. This is the case also for IL-6 (in Groups I and II) and one patient in Group II (patient with periprosthetic infection). These other mediators are not shown as there was only some individual increase of TNF*α* in OA-patients but no major difference between the different groups. Increased TH1 lineage commitment is reflected by increased IFN*γ* expression. Here we found marked IFN*γ* upregulation not only typically in the Ni-induced PT reactions, but in particular also in the Group I TKR patients, suggesting its role in periprosthetic disease progression. Interestingly, Jämsen and coworkers recently reported that they found scattered CD3+ T cells in the interface tissue of aseptically loosened hip arthroplasty with predominant macrophage related wear particle response. However, neither by quantitative PCR nor by immunohistochemistry they could show significant TH1 (namely, IFN*γ*) or TH2 (IL-4) mediator expression [36]. Since apart from IL-6 [37] in particular TGF-ß might play a central role in the onset and persistence of periprosthetic, articular fibrosis [38], we here analysed its respective expression in the different tissue samples. We observed an increase of TGF-ß expression in the metal sensitive TKR patients with however interindividual variations. Figures 3(a)–3(c) summarize these findings.

### 3.4. Comparison of Pre- and Postoperative WOMAC Score

**Figure 4:**
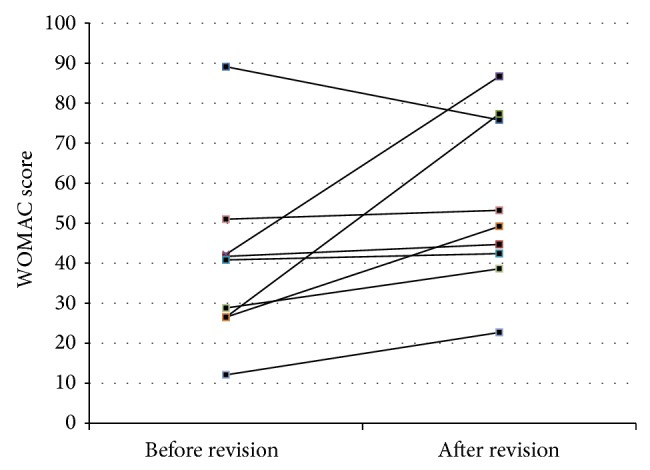
WOMAC score before and after revision surgery in 9 patients who received “hypoallergenic” material (8x titanium, 1x oxinium). The score-system has been used in accordance with the publication of Roos et al. [23].

19 of the 20 TKR patients responded to our request and completed a postoperative WOMAC scoring. 9 patients reported that at revision a “hypoallergenic” TKR had been implanted (8x Ti-based surface coating, 1x oxinium based implant). 8/9 patients did profit from this approach, as shown in Figure 4. So far there are only case reports or small patient series regarding the potential benefit from the use of “hypoallergenic” TKR [39, 40]. These results however stress the need of follow-up studies at a larger scale.

There are however limitations in the study: the facts that periprosthetic tissue samples may reflect only the actual stage of a dynamic process and that OA patients may not be as well a “control” as interface tissue probes from patients with well-functioning implants and the limited sample number in this investigations. Thus further studies are needed to validate the multimodular diagnostic approach in metal implant allergy.

## 4. Conclusions

This study demonstrates for the first time the potential of utilizing combined analytic steps to provide an approach to develop diagnostic characteristics of metal implant allergy. Allergy diagnostics (PT and LTT) and periprosthetic histology point to immunological response to implant alloy metals and the pattern of inflammatory mediator expression adds to functional differentiation.

Unexpected findings were the predominant “fibrotic” type IV interface response in the metal sensitized TKR patients and the marked IFN*γ* expression in the PT-positive TKR patients.

## Figures and Tables

**Table 1:** Patients characteristics.

	Sex	Age (years)	Tissue from	Implant survival (month)
Group I (revision surgery, patch test+, LTT+)	7 m 4 f	ø59.0 (37–75)	Knee	ø25.7 (6–30)
Group II (revision surgery, patch test−, LTT+)	7 m 2 f	ø64.2 (53–71)	Knee	ø28.0 (12–60)
Group III (revision surgery, patch test−, LTT−)	2 m 3 f	Ø69.8 (59–75	Knee	Ø23.4 (7–42)
OA-control Group (gonarthrosis before arthroplasty)	1 m 11 f	69.2 (52–89)	Knee	—
PT-control Group (patch test+ biopsies) (patch test− biopsies)	1 m 5 f 1 m 1 w	64.25 (53–75)	Back Back	— —

**Table 2:** Patch test/LTT results and histology grading, group I.

Patient number	Age, sex	Patch test reaction	LTT-reaction	CD3-infiltrate		KP1	CD20	CD15	Rating (Type I–IV according to [24])
				Qualitative	Quantitative				
10	37, m	Ni, Co, Cr	n.d.	Micronodal perivascular	+	++	−	−	Type 4
8	51, f	Ni,	Ni,	Micronodal perivascular	++	+	−	−	Type 4
15	59, f	Ni	n.d.	Micronodal perivascular	++	++	+	−	Type 4
9	74, m	Ni, Co	n.d.	Micronodal perivascular	+++	++	−	−	Type 4
3	51, m	Co	n.d.	Diffuse	−	+	−	−	Type 4
18	58, f	Ni,	Ni	Diffuse	−	−	−	−	Type 1 (Necrosis)
7	75, m	Ni	Ni	Diffuse	+	++	−	−	Type 4
1	63, m	Ni, Co, Ge, Be	Ni	Diffuse	+	++	+	+	Type 4
16	57, m	Ni, Co	Ni	Diffuse	+	+	−	−	Type 4
17	68, m	Ni, Co, Cr,	n.d.	Diffuse	+	+	−	−	Type 4
19	56, f	Ni,	neg	Diffuse	+	++	−	−	Type 1

Findings in 11 patients with CoCrMo based knee arthroplasty with complications and positive patch test reaction. Ni = nickel, Co = cobalt, Cr = chromium, Ge = gentamicin, B = benzoyl peroxide; n.d. = not done; LTT = lymphocyte transformation test.

**Table 3:** Patch test/LTT results and histology grading, Group II.

Patient number	Age, sex	Patch test reaction	LTT-reaction	CD3-infiltrate		KP1	CD20	CD15	Rating (Type I–IV according to [24])
				Qualitative	Quantitative				
11	61, m	neg	Ni	Diffuse	−	+	−	−	Type 4
12	65, f	neg	Ni	Diffuse	−	++	−	−	Type 1
14	66, m	neg	Ni, Co	Diffuse	−	++	−	−	Type 4
2	71, m	neg	Ni	Diffuse	+	++	+	−	Type 1
4	66, m	neg	Ni	Diffuse	+	+	−	−	Type 4
5	64, m	neg	Ni	Diffuse	+	+	−	−	Type 4
6	53 m	neg	Ni	Diffuse	+	++	−	−	Type 4
13	69, m	neg	Ni	Diffuse	+	−	−	−	Type 4
20	63, f	neg	Ni	Diffuse	+	++	−	+	Type 1

Findings in 9 patients with CoCrMo based knee arthroplasty with complications, negative patch test but positive lymphocyte transformation test (LTT); abbreviations see Table 2.

**Table 4:** Patch test/LTT results and histology grading, group III.

Patient number	Age, sex	Patch test reaction	LTT-reaction	CD3-infiltrate		KP1	CD20	CD15	Rating (Type I–IV according to [24])
				Qualitative	Quantitative				
IAR 6	59, f	neg	Neg	−	−	−	−	−	n.a.^*^
IAR 18	73, f	neg	Neg	−	−	−	−	−	Type 4
IAR 23	74, f	neg	Neg	−	−	−	−	−	Type 4
IAR 26	68, m	neg	Neg	−	−	−	−	+	Type 2
IAR 5	75, m	neg	Neg	Focal	+++	+++	+++	+++	Type 2

^*^n.a.: not applicable because of fibrinoid necrosis.

Findings in 5 patients with CoCrMo based knee arthroplasty with complications, negative patch test, and negative LTT; for abbreviations see Table 2.
